# Targeted urinary metabolomics combined with machine learning to identify biomarkers related to central carbon metabolism for IBD

**DOI:** 10.3389/fmolb.2025.1615047

**Published:** 2025-08-11

**Authors:** Miao-Lin Lei, Guan-Wei Bi, Xiao-Lin Yin, Yue Wang, Zi-Ru Sun, Xin-rui Guo, Hui-peng Zhang, Xiao-han Zhao, Feng Li, Yan-Bo Yu

**Affiliations:** ^1^ Department of Gastroenterology, Qilu Hospital, Shandong University, Jinan, Shandong, China; ^2^ Department of Gastroenterology, Qilu Hospital of Shandong University, Jinan, China; ^3^ Clinical Epidemiology Unit, Qilu Hospital of Shandong University, Jinan, China; ^4^ National Key Laboratory for Innovation and Transformation of Luobing Theory, Jinan, China; ^5^ The Key Laboratory of Cardiovascular Remodeling and Function Research, Chinese Ministry of Education, Chinese National Health Commission and Chinese Academy of Medical Sciences, Jinan, China; ^6^ Department of Cardiology, Qilu Hospital of Shandong University, Jinan, China; ^7^ Department of Pancreatic Surgery, General Surgery, Qilu Hospital of Shandong University, Jinan, China; ^8^ Department of Gastroentero-Pancreatic Surgery, Qilu Hospital (Qingdao), Cheeloo College of Medicine, Shandong University, Qingdao, Shandong, China

**Keywords:** inflammatory bowel disease, ulcerative colitis, Crohn’s disease, urinary metabolomics, machine learning, central carbon metabolism

## Abstract

**Introduction:**

Inflammatory bowel disease (IBD), comprising Crohn’s disease (CD) and ulcerative colitis (UC), is a chronic and relapsing inflammatory disorder of the gastrointestinal tract. Current diagnostic approaches are invasive, costly, and time-consuming, underscoring the need for non-invasive, accurate diagnostic methods.

**Methods:**

We conducted a targeted metabolomic analysis of 49 metabolites related to central carbon metabolism in urinary samples from individuals with IBD and control group. Diagnostic models were constructed using six machine learning algorithms, and their performance was evaluated by cross-validated area under the receiver operating characteristic curve (AUC). The SHAP (SHapley Additive exPlanations) method was used to interpret the models and identify key discriminatory features.

**Results:**

Six metabolites—xylose, isocitric acid, fructose, L-fucose, N-acetyl-D-glucosamine (GlcNAc), and glycolic acid—differentiated UC from control group, while three metabolites—xylose, L-fucose, and citric acid—distinguished CD from control group. The optimal diagnostic model achieved a mean AUC of 0.84 for UC and 0.93 for CD. These models retained high diagnostic accuracy even after adjusting for disease activity. SHAP analysis identified L-fucose, xylose, and GlcNAc as important features for UC, and citric acid and xylose for CD.

**Discussion:**

Our findings highlight distinct metabolic signatures in central carbon metabolism associated with IBD subtypes. The identified metabolite panels, combined with machine learning models, offer promising non-invasive tools for differentiating UC and CD from healthy individuals.

## Introduction

Inflammatory bowel disease (IBD) is a chronic and relapsing inflammatory disorder of the gastrointestinal tract, with ulcerative colitis (UC) and Crohn’s disease (CD) being the primary classifications. The incidence and prevalence of IBD are rapidly increasing, particularly in newly industrialized countries (Kaplan, 2015). IBD is typically diagnosed using standard clinical, endoscopic, radiological, and histological criteria (Rubin et al., 2019; Lichtenstein et al., 2018). Although UC and CD can present with similar clinical manifestations, their pathogenesis and treatments differ considerably (Bernstein et al., 2010; Blonski et al., 2012). Evidence has indicated that long-lasting subclinical disease activity usually reduces the quality of life and increases the risk of surgical intervention (Lichtenstein et al., 2004). Therefore, diagnosing and monitoring IBD disease activity are particularly crucial.

Currently, endoscopy examination is the gold standard method for IBD diagnosis, but it is time-consuming, invasive, and expensive (Hamilton, 2012; Annese et al., 2013). Some serum biomarkers, such as C-reactive protein (CRP), erythrocyte sedimentation rate (ESR), anti-Saccharomyces cerevisiae antibodies (ASCA), and perinuclear antineutrophil cytoplasmic antibodies (p-ANCA), are limited by their low sensitivity and specificity (Sands, 2015; Sakurai and Saruta, 2023).

Metabolomics offers a comprehensive analysis of metabolites, facilitating disease diagnosis and biomarker identification (Nicholson and Lindon, 2008). Urine metabolomics has emerged as a promising approach for identifying non-invasive biomarkers for disease diagnosis (Khamis et al., 2017). Central carbon metabolism, which includes glycolysis/gluconeogenesis, the pentose phosphate pathway, and the tricarboxylic acid (TCA) cycle, plays a crucial role in cellular function by providing energy and precursors for biosynthetic pathways (Wu et al., 2023; Xia et al., 2022). Extensive research has shown that some differential metabolites involved in central carbon metabolism, such as succinic acid and citric acid, differed significantly between IBD and healthy people (Schicho et al., 2012; Stephens et al., 2013; Dawiskiba et al., 2014; Alonso et al., 2016; Aldars-García et al., 2024; Martin et al., 2016; Martin et al., 2017). However, previous studies have often focused on single metabolites and lacked quantitative analysis of central carbon metabolism.

**FIGURE 1:**
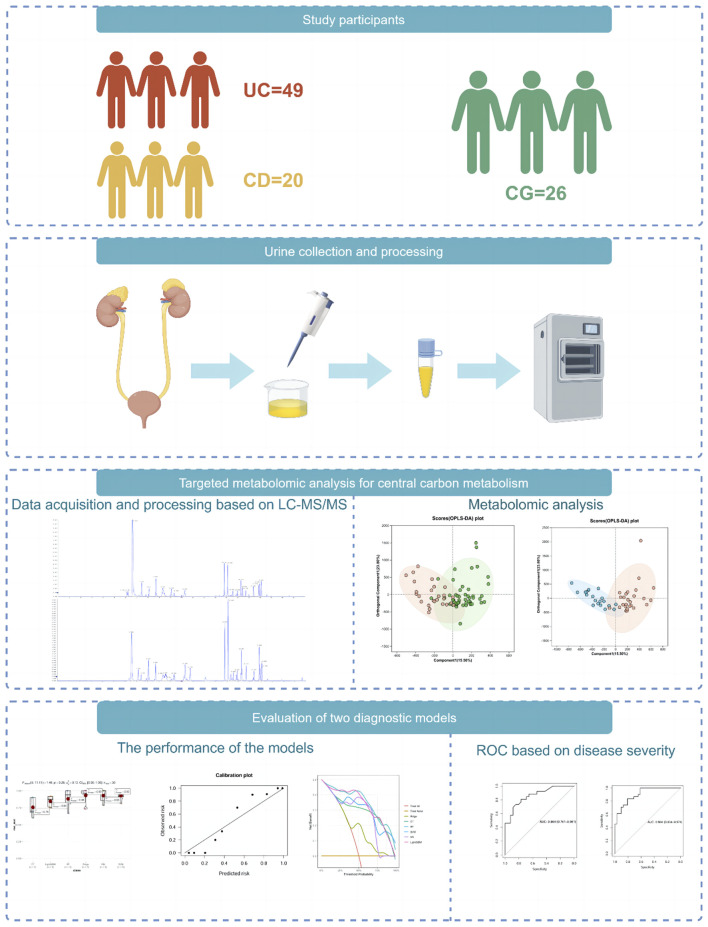
Overview of the study design. The schematic illustrates the overall workflow of the study, including participant recruitment (UC, CD, and control groups), urine sample collection and preparation, targeted metabolomic profiling using UHPLC-MS/MS, machine learning model construction and evaluation, and SHAP-based feature interpretation. (Drawn by Figdraw platform, ID: TTTIW05057).

In this study, we used ultra-high-pressure liquid chromatography coupled with tandem mass spectrometry (UHPLC-MS/MS) to perform a quantitative analysis of central carbon metabolism in urine samples collected from 95 subjects, including UC, CD, and control group (CG). We aimed to establish diagnostic models for discriminating IBD from non-IBD individuals using machine learning methods and evaluate the importance of metabolites. The overall study design is shown in Figure 1.

## Materials and methods

### Study participants

This study was approved by the Ethics Committee of Qilu Hospital of Shandong University (Approval number: KYLL-202212-010), and written informed consent was obtained from all subjects. A total of 49 UC patients, 20 CD patients, and 26 control subjects were recruited from hospitalized patients in the Qilu Hospital of Shandong University between April 2023 and June 2024. Eligible patients were between 18 and 75 years old, with a diagnosis of ulcerative colitis (UC) or Crohn’s disease (CD) confirmed according to the European Crohn’s and Colitis Organization criteria (Magro et al., 2017; Gomollón et al., 2017). Clinical activity was scored using the Mayo score for UC (Paine, 2014) and the Crohn’s disease activity index (CDAI) for CD (Best et al., 1976). The inclusion criterion for control group was healthy adults between 18 and 75 years of age.

To minimize inter-individual variation due to hydration status and recent dietary intake, all urine samples were collected as first-morning voids following an overnight fast of at least 8 h. Participants were instructed to avoid food and fluid intake after midnight and to collect their first urination immediately upon waking. This standardized collection helps ensure consistency in urinary metabolite concentrations by reducing the influence of short-term fluctuations in fluid balance and nutritional status.

To minimize possible confounding effects in the results, exclusion criteria were as follows: indeterminate colitis; structural abnormalities of the gastrointestinal tract; urinary dysfunction or infection; and combined with diabetes mellitus, inborn errors of metabolism, renal or hepatic disease, severe infection, or evidence of malignancy that could affect the results of this study. All participants adhered to the same exclusion criteria.

### Sample processing and preparation of standards

Fresh midstream urine samples were collected from subjects in the morning. Immediately after collection, the urine was centrifuged at 1,000 × *g* for 10 min to collect the supernatant. After centrifugation at 12,000 × *g* for 10 min at 4°C, the middle layer of the supernatant was aspirated into a 1.5 mL centrifuge tube and stored at −80°C until use.

A total of 49 central carbon metabolism-related standards were accurately weighed and individually dissolved in 50% methanol-water to prepare single-compound stock solutions. Appropriate volumes of each stock solution were combined and diluted with 50% methanol-water to obtain a mixed working standard solution at suitable concentrations.

Isotopically labeled internal standards—including succinic acid-D4, L-carnitine-D3, cholic acid-D4, and salicylic acid-D4—were also weighed and dissolved individually in 50% methanol-water to prepare their respective stock solutions. Equal volumes of these solutions were then combined and diluted to prepare a mixed internal standard solution with final concentrations of 5 μg/mL (succinic acid-D4), 5 μg/mL (L-carnitine-D3), 20 μg/mL (cholic acid-D4), and 30 μg/mL (salicylic acid-D4).

To construct the calibration curves, 50 μL of the working standard solution was mixed with 10 μL of the isotope internal standard mixture and 140 μL of acetonitrile. The mixture was vortexed for 1 min and centrifuged at 14,000 rcf for 20 min at 4°C. Then, 100 μL of the resulting supernatant was transferred to a 1.5 mL centrifuge tube, followed by the addition of 25 μL of 200 mM 3-nitrophenylhydrazine hydrochloride (3NPHHCl) and 25 μL of 120 mM EDCHCl solution containing 6% pyridine. The mixture was vortexed for 30 s, briefly centrifuged for 5 s, and incubated at 60°C for 40 min using a thermostatic shaker. This reaction forms hydrazone derivatives with carbonyl-containing metabolites, which is known as derivatization. After the reaction, the sample was vortexed again for 30 s, centrifuged at 14,000 rcf for 20 min at 4°C, and the supernatant was transferred into an autosampler vial for LC-MS/MS analysis. Calibration curves for all 49 central carbon metabolites were constructed using 12 concentration points, each of which was injected in triplicate. The resulting peak area ratios (analyte/internal standard) were used to generate calibration curves through weighted linear regression. Detailed infromation were listed in Supplementary Table S5.

A 20 μL aliquot of urine was mixed with 10 μL of isotope internal standard, 30 μL of 50% methanol, and 140 μL of acetonitrile, and vortexed for 1 min. After centrifugation at 14,000 rcf for 20 min at 4°C, 100 μL of the supernatant was transferred to 1.5 mL centrifuge tubes containing 25 μL of 200 mM 3NPH.HCL and 25 μL of 120 mM EDC. HCL (containing 6% pyridine) solution. The mixture was shaken in a vortex for 30 s, centrifuged for 5 s, and then reacted at 60°C for 40 min in a thermostatic oscillator. After the reaction, the mixture was vortexed for 30 s and centrifuged at 14,000 rcf for 20 min at 4°C. Finally, the supernatant was transferred to an injection vial for subsequent analysis.

### UHPLC-MS/MS analysis

Targeted metabolomic analysis of the urine samples was performed using liquid chromatography–tandem mass spectrometry (LC-MS/MS) on an ExionLC AD system coupled with a QTRAP® 6500+ mass spectrometer (Sciex, United States) at Majorbio Bio-Pharm Technology Co. Ltd. (Shanghai, China).

Chromatographic separation was performed on an ExionLC™ AD system equipped with a Waters HSS T3 chromatography column (2.1 × 150 mm, 1.8 μm). Mobile phase A was 0.03% formic acid in water, and mobile phase B was 0.03% formic acid in methanol. The gradient was as follows: 0.0–2.0 min, hold at 1% B; 2.0–8.0 min, from 1% to 22% B; 8.0–12.0 min, hold at 22% B; 12.0–13.0 min, from 22% to 40% B; 13.0–17.0 min, from 40% to 65% B; 17.0–19.0 min, hold at 65% B; 19.0–20.0 min, from 65% to 100% B; 20.0–21.0 min, hold at 100% B; 21.0–21.01 min, from 100% to 1% B; 21.01–22.0 min, hold at 1% B. The injection volume was 2 μL, and the column temperature was 40°C.

Mass spectrometric analyses were performed on a QTRAP® 6500+ mass spectrometer (Sciex, United States) equipped with an electrospray ionization (ESI) source operating in negative and positive modes. The parameters were set as follows: source temperature (TEM) at 550°C; curtain gas (CUR) at 35 psi; collision gas (CAD) at medium; both Ion Source Gas1 and Gas2 at 55 psi; IonSpray Voltage (IS) at +4500/−4500 V.

Data acquisition was conducted in multiple reaction monitoring (MRM) mode, a highly sensitive and specific mass spectrometric technique that enables the selective quantification of predefined metabolites. In MRM mode, the mass spectrometer first selects precursor ions (parent ions) of interest in the first quadrupole (Q1), induces fragmentation through collision-induced dissociation (CID) in the second quadrupole (Q2), and then monitors specific product ions in the third quadrupole (Q3). This approach ensures high analytical specificity, sensitivity, and reproducibility for the targeted detection and quantification of known metabolites. Metabolite identification was carried out in a targeted manner using MRM based on authentic reference standards. Both precursor and corresponding product ions were monitored for each metabolite, enabling high-confidence identification. The complete list of ion pairs and retention time data is provided in Supplementary Table S3.

The quality control (QC) sample was a mixed standard solution at a moderate concentration level, primarily used to evaluate the stability of the analytical system. In this study, the QC sample was prepared using the C10 concentration level (i.e., the 10th calibration level, see Supplementary Table S5). During the LC-MS/MS analysis, one QC sample was injected after every 5 to 10 sample injections to monitor instrument stability and repeatability. The stability of QC signals across the analytical batch further validated the reproducibility of the instrument and analytical conditions. The relative standard deviations (RSDs) of all target analytes were below 15%, indicating that the method and analytical system were stable and reliable.

To evaluate the robustness of the LC-MS/MS platform and exclude the potential influence of instrumental variation, we conducted method validation for 49 central carbon metabolites. QC samples at low, medium, and high concentrations were analyzed in six replicates within a single day and across 3 days. Intra-day and inter-day precision were calculated as RSD%, and recovery rates were calculated based on spiked and measured concentrations. Acceptable thresholds were defined as RSD% ≤15% and recovery rates within 80%–120%, following common metabolomics validation guidelines. All metabolites met these criteria, confirming the stability of the measurement system.

The raw data were processed by Sciex software OS by using the default parameters and assisting manual inspection. A linear regression standard curve was created with the ratio of the mass spectral peak area of the analyte to the internal standard peak area as the vertical coordinate and the concentration of the analyte as the horizontal coordinate. The ratio of the mass spectral peak area of the analyte to the internal standard peak area was substituted into the linear equation to calculate the sample’s concentration (Supplementary Tables S1, S2).

### Statistical analysis

SPSS version 27.0.1 and R software (version 4.4.2) were used to process and analyze clinical and metabolomic data. Normally distributed data were expressed as mean ± standard deviation and compared using a t-test. Non-normally distributed data were expressed as median and interquartile ranges and analyzed using the Mann-Whitney U-test. Qualitative data were presented using frequencies and percentages and compared with the Chi-square test. Multivariate statistical analysis was performed using principal component analysis (PCA), partial least squares discriminant analysis (PLS-DA), and orthogonal partial least squares discriminant analysis (OPLS-DA) to identify differential metabolites and visualized by the ggplot2 package. Seven-fold cross-validation and permutation tests were used to evaluate the quality of the model. Enrichment of Kyoto Encyclopedia of Genes and Genomes (KEGG) pathways was also performed. Multivariable logistic regression, adjusting for age, sex, smoking, weight, and height, was used to evaluate the association of each metabolite with disease status. Metabolites with a VIP >1 in OPLS-DA analysis and Padj values <0.05 were defined as significantly differential metabolites.

### Machine learning models

Machine learning operations were performed using the “tidymodels” package. The SMOTE algorithm was utilized to address unbalanced class distribution issues in the models. Several optimized machine learning models were used to discriminate between IBD and control group: decision tree (DT), random forest (RF), ridge regression (Ridge), support vector machine (SVM), light gradient boosting machine (LightGBM), and neural networks (NN). The hyperparameters were obtained using grid search with 5-fold cross-validation. The performance of these machine learning methods was evaluated by the area under the receiver operating characteristic curve (AUC), calibration curves, and decision curve analysis (DCA). We also utilized Shapley additive explanation (SHAP) values to improve the interpretability of the final model.

## Results

### Participant characteristics

A total of 95 participants were enrolled in this study, including 49 patients with ulcerative colitis (UC), 20 patients with Crohn’s disease (CD), and 26 control individuals. The clinical characteristics of the three groups are summarized in Table 1. Statistically significant differences were observed in age and weight between the IBD groups and the control group (p < 0.01), as well as in hematological parameters such as hemoglobin, hematocrit, and platelet count. These differences highlight the clinical and physiological alterations associated with IBD, which may be reflected in the metabolomic profiles.

**TABLE 1:** Clinical characteristics of the study participants.

Variables	Ulcerative colitis (n = 49)	Crohn’s disease (n = 20)	Control group (n = 26)
Sex, n (%)			
Male	35 (71.43)	12 (60.00)	17 (65.38)
Female	14 (28.57)	8 (40.00)	9 (34.62)
Age, (years)	47.59 ± 15.99	40.20 ± 17.75**	53.54 ± 13.94
Weight, (kg)	61.54 ± 10.49**	59.40 ± 10.56**	71.02 ± 12.35
Height, (m)	1.70 ± 0.07	1.69 ± 0.10	1.68 ± 0.07
Smoke, n (%)			
Yes	16 (32.65)	6 (30.00)	10 (38.46)
No	33 (67.35)	14 (70.00)	16 (61.54)
WBC, (10^9^/L)	7.40 ± 2.58**	5.55 ± 2.04	6.12 ± 1.36
Hb, (g/L)	121.00 (106.50,135.50)***	117.50 (95.75,137.75)**	140.25 (133.00,148.25)
Hct, (%)	38.60 (34.00,41.85)**	35.85 (30.20,41.10)**	41.84 (40.20,43.93)
PLT, (10^9^/L)	297.00 (217.00,421.00)**	217.00 (165.25,252.5)	225.65 (213.25,240.25)
Extent UC, n (%)			
Ulcerative proctitis (E1)	9 (18.37)		
Distal colitis (E2)	11 (22.45)		
Extensive colitis (E3)	29 (59.18)		
Location CD, n (%)			
Ileal (L1)		12 (60.00)	
Colonic (L2)		4 (20.00)	
Ileocolonic (L3)		4 (20.00)	
Upper GI (L4)		2 (10.00)	
Disease activity, n (%)			
Remission	9 (18.37)	10 (50.00)	
Mild	9 (18.37)	3 (15.00)	
Moderate	20 (40.82)	7 (35.00)	
Severe	11 (22.45)		

Abbreviation: WBC, white blood count; PLT, platelet; RBC, red blood count; Hb, hemoglobin; Hct, hematocrit.

*P < 0.05, **P < 0.01, ***P < 0.001 compared with CG.

### Metabolomic profiling and multivariate analysis

**FIGURE 2:**
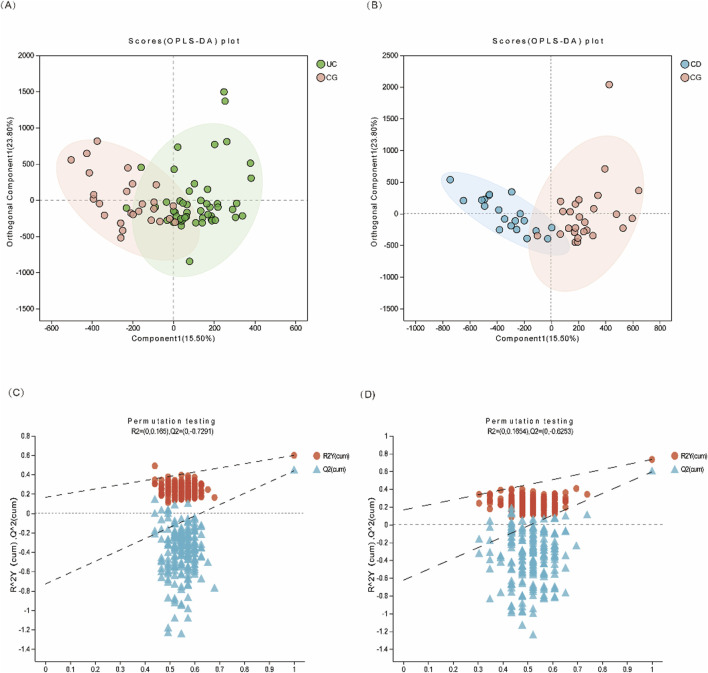
Multivariate statistical analysis of urinary metabolites in IBD patients. **(A)** OPLS-DA score plot of UC (green dots) vs. CG (pink dots); **(B)** OPLS-DA score plot of CD (blue dots) vs. CG (pink dots). **(C, D)** The 200-time permutation plots of two OPLS-DA models, respectively.

Targeted metabolomic profiling identified 49 urinary metabolites associated with central carbon metabolism across all subjects. Principal component analysis (PCA) showed limited separation among the three groups, indicating the necessity for supervised methods. Orthogonal partial least squares discriminant analysis (OPLS-DA) revealed clear group separation between UC and controls (*R* ^2^X = 0.906, *R* ^2^Y = 0.598, *Q* ^2^ = 0.439), and between CD and controls (*R* ^2^X = 0.543, *R* ^2^Y = 0.733, *Q* ^2^ = 0.594), suggesting that metabolite patterns differ significantly between disease and non-disease states (Figure 2, Supplementary Figure S1).

**FIGURE 3:**
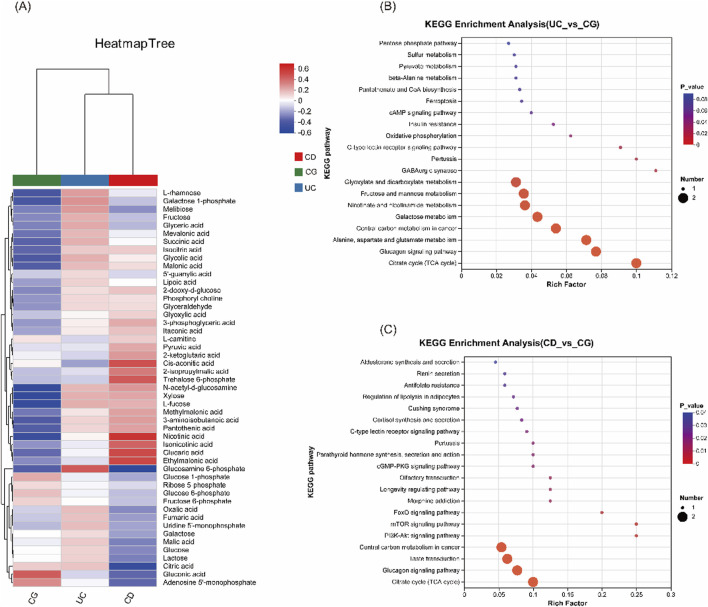
Comparison of urinary metabolomic profiles and KEGG pathway enrichment analysis. **(A)** Heatmap of cluster analysis of each metabolite among the three groups (UC, CD and CG), illustrating differential metabolite abundance. **(B)** KEGG enrichment analysis between UC vs CG. **(C)** KEGG enrichment analysis between CD vs CG.

Hierarchical clustering of metabolite intensities showed distinct metabolic signatures among UC, CD, and control groups (Figure 3A). Metabolites such as glucosamine-6-phosphate were elevated in UC, whereas cis-aconitic acid, trehalose-6-phosphate, nicotinic acid, and glucaric acid were more abundant in CD compared to both UC and control groups. KEGG pathway enrichment analysis further supported that metabolic pathways including the TCA cycle, glucagon signaling, PI3K-Akt, and mTOR signaling were perturbed in IBD, suggesting potential disease-specific metabolic reprogramming (Figures 3B,C).

### Identification of differential metabolites and diagnostic panels

**FIGURE 4:**
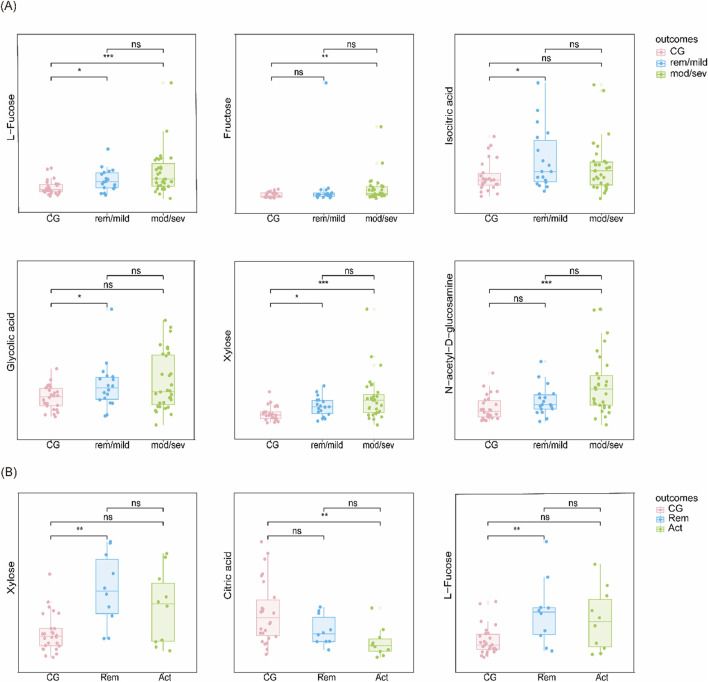
Candidate biomarkers based on disease severity **(A)** Metabolite levels in UC vs CG stratified by disease severity (remission/mild vs moderate/severe). **(B)** Metabolite levels in CD vs CG stratified similarly.

Logistic regression models adjusting for age, sex, smoking status, weight, and height identified several significantly differential metabolites. Six metabolites—xylose, isocitric acid, fructose, L-fucose, GlcNAc, and glycolic acid—were significantly altered in UC patients compared to controls. In CD, three metabolites—xylose, L-fucose, and citric acid—were significantly changed. Notably, L-fucose and xylose were elevated in both UC and CD, highlighting their potential as shared biomarkers. The differences in metabolite levels were more pronounced in patients with higher disease activity, as shown in Figure 4.

To assess the potential impact of instrumental variation, we performed method validation based on intra- and inter-day precision as well as recovery rate analyses. A total of 49 central carbon metabolites were evaluated using quality control (QC) samples prepared at three concentration levels (low, medium, and high). Each level was analyzed in six replicates within a day (intra-day precision) and across 3 days (inter-day precision). The RSD of intra-day precision ranged from 1.20% to 11.92%, and that of inter-day precision ranged from 2.33% to 12.66%. Recovery rates ranged from 85.33% to 113.71%. These results confirm that the analytical platform is highly stable and reproducible under the current workflow. Therefore, the observed metabolite differences are unlikely to be attributed to instrumental drift. The detailed information was listed in Supplementary Table S4.

### Machine learning algorithms

**FIGURE 5:**
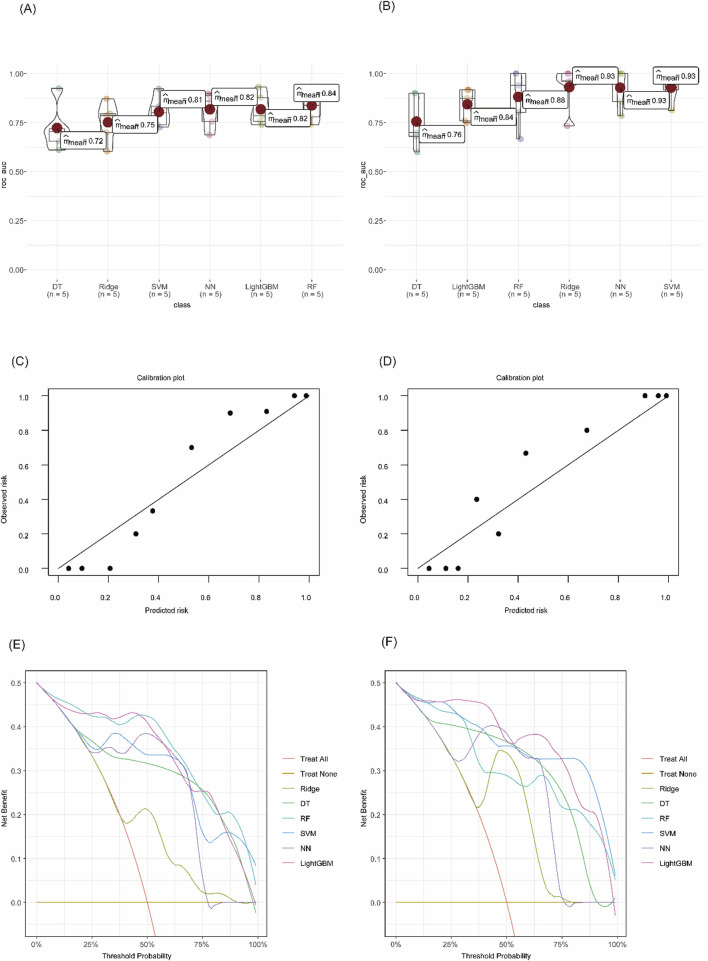
Evaluation of diagnostic model performance using machine learning algorithms. **(A)** Cross-validated AUC values for each model in UC vs CG. **(B)** Cross-validated AUC values in CD vs CG. **(C)** Calibration curve of the RF model for UC. **(D)** Calibration curve of the SVM model for CD. **(E)** DCA of different models in distinguishing UC from CG. **(F)** DCA curves for CD vs CG.

Six machine learning algorithms were trained to distinguish IBD subtypes from controls. Among them, the random forest (RF) model achieved the best performance for UC, with a mean cross-validated AUC of 0.84 (SE = 0.036), and the support vector machine (SVM) model performed best for CD, with a mean AUC of 0.93 (SE = 0.035). Calibration curves showed good model fit, and decision curve analysis (DCA) demonstrated that both models offered higher net clinical benefit compared to baseline strategies (Figures 5A–F).

**FIGURE 6:**
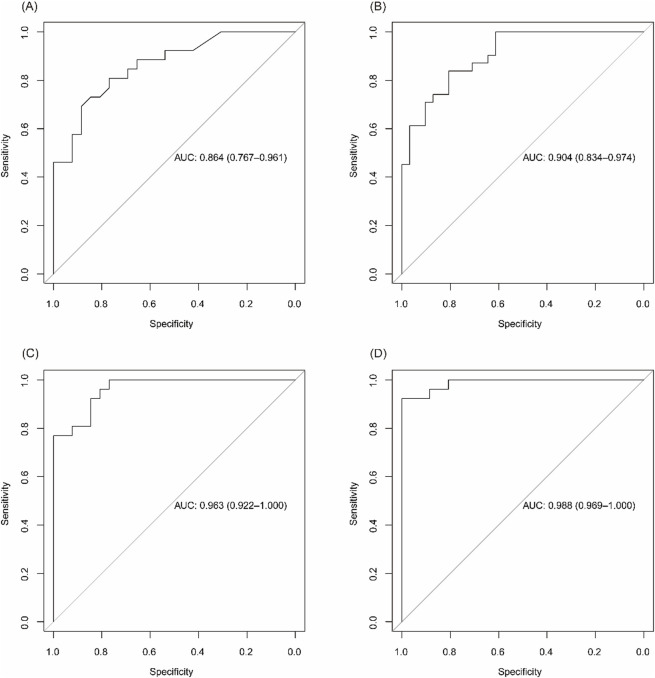
Diagnostic performance of biomarker panels across disease activity levels. **(A)** ROC curve for identifying UC patients in remission/mild activity vs CG. **(B)** ROC curve for moderate/severe UC vs CG. **(C)** ROC curve for CD patients in remission vs CG. **(D)** ROC curve for active CD vs CG.

Stratified analysis by disease activity showed that the diagnostic accuracy remained high in both mild/remission and moderate/severe stages. The AUC for distinguishing UC from controls was 0.864 in remission/mild patients and 0.904 in moderate/severe cases. Similarly, CD patients showed AUCs of 0.963 in remission and 0.988 in active disease, confirming the robustness of the models across disease stages (Figures 6A–D).

**FIGURE 7:**
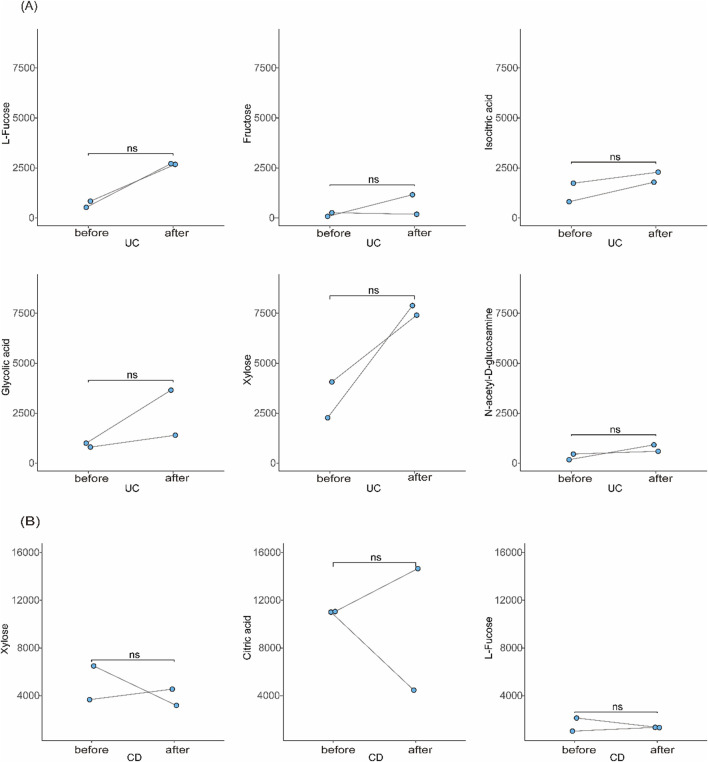
Stability of candidate biomarkers over time. **(A)** Longitudinal comparison of urinary metabolite levels in UC patients between two time points ≥6 months apart with similar disease activity. **(B)** Similar analysis in CD patients.

Longitudinal data from a subset of patients with samples collected at ≥6-month intervals showed consistent metabolite profiles during periods of equivalent disease activity, indicating good temporal stability of the biomarkers (Figure 7).

### Model interpretation using SHAP

**FIGURE 8:**
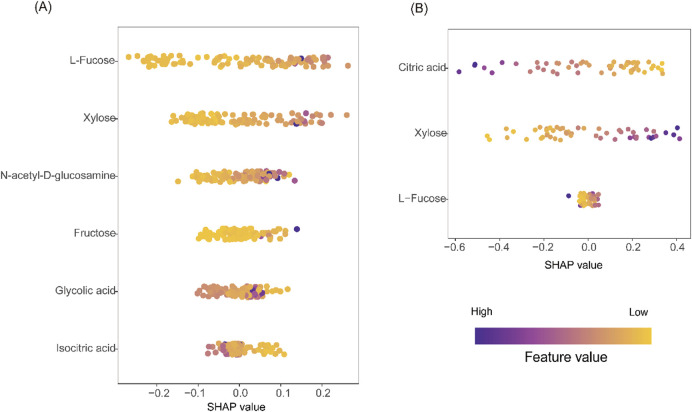
Model interpretation using SHAP analysis. **(A)** SHAP beeswarm plot from the RF model for UC vs CG. **(B)** SHAP beeswarm plot from the SVM model for CD vs CG. In both plots, purple indicates higher feature values, yellow indicates lower values. Positive SHAP values on the X-axis correspond to increased predicted disease risk; negative values indicate lower risk. The vertical axis ranks features by overall contribution to model output.

To interpret the contributions of individual metabolites to model predictions, SHAP (Shapley Additive exPlanations) analysis was performed (Lundberg and Lee, 2017). In the RF model for UC, L-fucose, xylose, and GlcNAc were identified as the top predictors, with positive SHAP values indicating higher disease risk (Figure 8A). For CD, citric acid and xylose were most influential in the SVM model, with decreased citric acid and elevated xylose contributing to increased CD probability (Figure 8B). These findings confirm the biological relevance and diagnostic utility of the selected metabolites.

## Discussion

To our knowledge, this is the first targeted urinary metabolomics study to explore the association between IBD and central carbon metabolism. In this study, we demonstrated that certain urine metabolites related to central carbon metabolism differ significantly between IBD patients and control group. Through machine learning algorithms, we also identified two potential biomarker panels to distinguish UC from control group, and CD from control group, respectively. Furthermore, they have value for patients with IBD at different stages. Overall, our research showed that the combined application of urinary metabolomics and machine learning had unique advantages in diagnosing and monitoring IBD disease activity.

The levels of xylose and L-fucose were significantly increased in both UC and CD compared with the control group, as reported in a previous study (Schicho et al., 2012; Murdoch et al., 2008). The changes in monosaccharides were unclear and might be related to dysbiosis in IBD. Xylose and L-fucose are metabolites related to intestinal flora; their production is inseparable from the involvement of intestinal flora. The enrichment of some bacterial genera [such as Actinobacteria (Manichanh et al., 2012) and *Streptococcus* (Scanu et al., 2024)] in UC may cause increased production and urinary excretion of xylose (Beg et al., 2001) and L-fucose (Moya-Gonzálvez et al., 2022). Increased production and urinary excretion of xylose (Robert and Bernalier-Donadille, 2003) and L-fucose (Moya-Gonzálvez et al., 2022) may be due to the enrichment of some bacterial genera [such as *Enterococcus* (Kang et al., 2010) and Ruminococcus (Manichanh et al., 2012)] in CD. Meanwhile, fucose itself is a critical component of mucin in the intestinal epithelial barrier and maintains intestinal flora homeostasis (Bets et al., 2022). Fujii H et al. further explored the underlying mechanism of fucose in IBD (Fujii et al., 2016). They discovered that the core-fucosylated T-cell receptor was essential for T-cell signaling and the production of inflammatory cytokines. Interestingly, recent research found that these monosaccharides and some polysaccharides containing them could reduce colon inflammation (Li et al., 2021; Hui et al., 2024; Lean et al., 2015; Qin et al., 2022). Exogenous L-fucose improved intestinal epithelial barrier function, and the mechanism was related to the upregulation of fucosyltransferase 2-mediated fucosylation of intestinal epithelial cells (Li et al., 2021). And L-fucose also improved the epithelial barrier by promoting the proliferation of intestinal stem cells (Tan et al., 2022). A polysaccharide containing xylose and fucose could significantly decrease Akkermansia to upregulate thiamine metabolism, thereby inhibiting macrophage activation and reducing oxidative stress and inflammation (Hui et al., 2024).

N-acetyl-D-glucosamine (GlcNAc), an amide derivative of the monosaccharide glucose, presents in parts of glycosaminoglycans, glycoproteins, and glycolipids (Chen et al., 2010; Das et al., 2024). Notably, GlcNAc is also a component of mucin and helps maintain the mucus barrier. Currently, the relationship between GlcNAc and UC has not yet been fully explored. Previous research indicated that GlcNAc could reduce the colonization of pathogenic bacteria, enhance the growth of beneficial bacteria, and elevate the expression of the tight junction protein occludin to improve intestinal barrier function (Choi et al., 2023; Jenior et al., 2017). Zhao M et al. discovered that reduced O-linked-N-acetylglucosaminylation (O-GlcNAcylation) levels lead to increased intestinal permeability and microbial imbalance in the gut in patients with UC (Zhao et al., 2018). UC is usually accompanied by intestinal barrier disruption and infiltration of immune cells. Matos I et al. confirmed that N-acetylglucosaminidase, a biomarker of macrophage infiltration, was increased in the colon of DSS-induced colitis mice (Matos et al., 2013). We thus speculated that the elevated levels of GlcNAc in the urine of UC patients may be associated with intestinal barrier disruption and increased N-acetylglucosaminidase. Supplementation with GlcNAc has been used to treat children with treatment-resistant IBD, with a significant improvement in symptoms (Salvatore et al., 2000).

Citric acid is an essential metabolite in the TCA cycle. The urinary citric acid levels were decreased in CD patients, especially in those patients with high levels of disease activity. This result was consistent with previous results (Schicho et al., 2012; Stephens et al., 2013; Dawiskiba et al., 2014; Alonso et al., 2016; Aldars-García et al., 2024). Chronic diarrhea may contribute to hypocitraturia in CD patients because of metabolic acidosis from a loss of bicarbonate in the feces. This may explain the increased risk of urinary stones in CD patients (Rudman et al., 1980; Siener et al., 2024). Citrate and hydroxycinnamate derivatives from Mume Fructus could relieve LPS-induced intestinal epithelial cell injury by regulating the FAK/PI3K/AKT signaling pathway and thus should be considered as potential therapeutic targets for CD (Liu et al., 2023).

As mentioned above, changes in the urine metabolic profile of IBD patients in our study were consistent with the findings of previous studies. Our research further employed machine learning techniques to evaluate the importance of the metabolites associated with central carbon metabolism. Finally, we built two potential biomarker panels to diagnose IBD, which showed good diagnostic performance even in different disease stages. ESR and CRP are the most widely used blood markers for IBD in clinical practice (Sands, 2015). The AUC values of CRP and ESR in UC patients were 0.607 and 0.552, respectively. In CD patients, the AUC values for CRP and ESR were 0.698 and 0.746, respectively (Huang et al., 2023). The AUC values of the potential biomarker panels were significantly greater than those of CRP and ESR, suggesting that the potential biomarker panels had higher accuracy in diagnosing IBD than these traditional indicators.

Several previous studies have explored urinary metabolomic changes in patients with IBD, predominantly using untargeted approaches such as NMR or global LC-MS. For instance, Schicho et al. and Stephens et al. demonstrated that urinary metabolites such as hippurate and citrate could distinguish IBD patients from healthy individuals, although pathway specificity was limited (Schicho et al., 2012; Stephens et al., 2013). Previous studies by Alonso et al. and Aldars-García et al. examined the urinary metabolome in immune-mediated or treatment-naïve IBD populations; however, these analyses primarily relied on non-targeted or global profiling approaches rather than quantitative methods. (Alonso et al., 2016; Aldars-García et al., 2024).

In contrast, our study employed a targeted metabolomic strategy focusing specifically on central carbon metabolism, enabling precise quantification of 49 metabolites involved in glycolysis, the TCA cycle, and related pathways. Moreover, we integrated multiple machine learning algorithms and SHAP interpretability analysis, which were not typically applied in previous urinary metabolomics studies for IBD. While some of our findings, such as reduced urinary citrate in CD and elevated fucose in active IBD, are consistent with previous observations (Schicho et al., 2012; Dawiskiba et al., 2014), the persistent elevation of xylose and GlcNAc in both UC and CD and their stability over time may offer novel diagnostic insights.

Compared to earlier untargeted studies, our quantitatively validated metabolite panel shows higher diagnostic performance and better clinical translation potential. These findings both confirm and expand upon prior research by identifying robust, pathway-relevant biomarkers for the non-invasive diagnosis and stratification of IBD.

While this study has some strengths, it also has some limitations. First, our study enrolled a relatively small sample of participants from a single institution. Therefore, large-scale and multicenter studies should be carried out to verify the robustness of the models. Second, we did not evaluate the effects of diet on urine metabolites. Morning urine samples were collected to minimize the impact of diet and physical activity. Third, we adjusted for several potential confounders, but residual confounding cannot be completely ruled out, moreover, sample randomization was not achieved during the targeted metabolomics analysis, which may have introduced potential bias. Fourth, we could not completely establish the stability of urine metabolites in this study. The quantitative determination of metabolites was performed in the same batch to reduce variability. The relative standard deviation of the stability of these targets was less than 15%, indicating that the results obtained by the method should be reliable. The precision and recovery were determined by analyzing high, medium, and low standard concentrations. Inter-day and intra-day precision was <15% and the recoveries were greater than 80% at all concentrations. Although our research evaluated the stability of urinary metabolites, the sample size was too small. Furthermore, longitudinal stability of metabolites was assessed in some studies and the results were credible (Khamis et al., 2017).

As future work, more participants, updated models and more key metabolic pathways are needed.

## Conclusion

In conclusion, this study identified a distinct panel of urinary metabolites related to central carbon metabolism that can accurately differentiate ulcerative colitis (UC) and Crohn’s disease (CD) from control individuals. By integrating targeted metabolomics with multiple machine learning algorithms, we developed robust diagnostic models with high predictive performance (AUC >0.90 for CD, and >0.80 for UC), even across different stages of disease activity. Key metabolites such as xylose, L-fucose, GlcNAc, and citric acid were found to be strongly associated with IBD status and may reflect underlying metabolic dysregulation in disease pathogenesis.

These findings not only validate previous metabolic signatures reported in IBD but also expand upon them by providing pathway-specific, quantitatively validated biomarker panels. Our results support the potential utility of urinary metabolite-based models as non-invasive diagnostic tools for IBD, offering a promising supplement or alternative to current invasive methods. Future large-scale and multicenter studies are warranted to further validate the generalizability and clinical applicability of these metabolite-based diagnostic strategies.

## Data Availability

The original contributions presented in the study are included in the article/Supplementary Material, further inquiries can be directed to the corresponding author.
