# Supplementary material for: Targeted urinary metabolomics combined with machine learning to identify biomarkers related to central carbon metabolism for IBD
Source: Front Mol Biosci. 2025 Aug 11;12:1615047. doi: 10.3389/fmolb.2025.1615047 (PMC12375463; doi:10.3389/fmolb.2025.1615047)
Supplement: Supplementary file 1 [file Table1.docx]

Table S1. Metabolite differences between UC and control group (CG).

| Metabolite | UC | CG | VIP | P | Padj |
| --- | --- | --- | --- | --- | --- |
| 2-deoxy-d-glucose | 1222.10 (974.15, 2669.69) | 1417.06 (800.53, 1946.46) | 0.417344 | 0.29 | 0.05 |
| 2-isopropylmalic acid | 37.75 (19.27, 112.94) | 13.60 (11.51, 23.76) | 0.080207 | <.001 | 0.961 |
| 2-ketoglutaric acid | 6604.08 (3729.01, 11359.73) | 7616.52 (4249.13, 12536.18) | 0.262259 | 0.525 | 0.332 |
| 3-aminoisobutanoic acid | 16452.00 (3382.51, 79020.25) | 5314.07 (1657.85, 24138.93) | 2.123756 | 0.085 | 0.432 |
| 3-phosphoglyceric acid | 1109.09 (496.46, 1921.54) | 706.08 (520.41, 1196.12) | 0.116111 | 0.15 | 0.078 |
| 5'-guanylic acid | 4.87 (3.24, 9.76) | 4.23 (3.03, 7.50) | 0.008441 | 0.646 | 0.256 |
| Adenosine 5'-monophosphate | 113.46 (77.50, 174.83) | 133.39 (75.72, 212.53) | 0.057751 | 0.251 | 0.478 |
| Cis-aconitic acid | 34536.98 (17974.46, 48016.94) | 44453.06 (26554.47, 60185.30) | 0.878637 | 0.081 | 0.704 |
| Citric acid | 190010.60 (120230.15, 385328.61) | 232073.59 (131585.43, 332350.03) | 0.225538 | 0.468 | 0.36 |
| Ethylmalonic acid | 1383.30 (944.79, 1981.87) | 1237.87 (636.57, 1639.62) | 0.183825 | 0.09 | 0.045 |
| Fructose | 4429.16 (3133.46, 9039.02) | 2323.17 (1374.36, 6034.46) | 1.216074 | 0.007 | 0.006 |
| Fructose 6-phosphate | 141.56 (119.24, 217.38) | 163.78 (111.71, 237.74) | 0.012643 | 0.554 | 0.409 |
| Fumaric acid | 350.06 (155.53, 739.36) | 332.69 (167.98, 604.26) | 0.115421 | 0.736 | 0.125 |
| Galactose | 5082.01 (3100.43, 8316.78) | 3310.66 (2403.64, 5527.36) | 0.046379 | 0.06 | 0.441 |
| Galactose 1-phosphate | 124.66 (91.73, 171.23) | 76.83 (57.53, 107.10) | 0.115989 | <.001 | 0.003 |
| Glucaric acid | 3541.60 (2188.12, 5416.63) | 2675.87 (1744.53, 4107.68) | 0.356797 | 0.135 | 0.125 |
| Gluconic acid | 77062.41 (48547.89, 136477.29) | 152736.32 (57067.21, 358477.58) | 4.040735 | 0.029 | 0.314 |
| Glucosamine 6-phosphate | 15.95 (12.84, 19.12) | 11.76 (10.91, 14.85) | 0.027913 | <.001 | ＜0.001 |
| Glucose | 22367.10 (12808.81, 45043.60) | 19736.87 (9644.03, 32298.40) | 1.172093 | 0.275 | 0.285 |
| Glucose 1-phosphate | 86.99 (31.58, 213.35) | 100.41 (44.56, 329.82) | 0.157065 | 0.678 | 0.723 |
| Glucose 6-phosphate | 253.15 (119.26, 549.55) | 223.04 (133.25, 380.03) | 0.140718 | 0.429 | 0.859 |
| Glyceraldehyde | 306.77 (175.45, 483.88) | 225.12 (167.44, 330.67) | 0.197883 | 0.17 | 0.024 |
| Glyceric acid | 2363.71 (1355.85, 3975.36) | 1267.49 (820.31, 2133.33) | 0.616183 | 0.022 | 0.012 |
| Glycolic acid | 18177.24 (11930.90, 27042.66) | 15191.49 (10408.06, 19504.50) | 1.56903 | 0.031 | 0.003 |
| Glyoxylic acid | 320.11 (198.89, 425.18) | 287.79 (224.32, 453.00) | 0.084208 | 0.769 | 0.277 |
| Isocitric acid | 23352.59 (13191.30, 32376.05) | 16146.56 (12225.60, 21714.61) | 1.294846 | 0.054 | 0.023 |
| Isonicotinic acid | 0.84 (0.44, 1.54) | 0.72 (0.30, 1.06) | 0.008799 | 0.162 | 0.036 |
| Itaconic acid | 284.33 (131.69, 408.02) | 199.95 (126.45, 280.79) | 0.065347 | 0.361 | 0.115 |
| Lactose | 7523.89 (5019.70, 12883.98) | 6530.46 (2506.34, 16316.06) | 0.375038 | 0.786 | 0.954 |
| L-carnitine | 7850.93 (2165.81, 20796.27) | 10824.00 (6497.61, 58137.20) | 0.727735 | 0.135 | 0.42 |
| L-fucose | 18243.54 (10802.51, 28784.89) | 8578.86 (6501.16, 13691.53) | 1.940287 | <.001 | 0.002 |
| Lipoic acid | 5.23 (3.27, 8.69) | 5.12 (2.03, 7.28) | 0.009918 | 0.216 | 0.13 |
| L-rhamnose | 496.57 (281.64, 696.74) | 326.12 (213.24, 380.73) | 0.294045 | 0.008 | 0.053 |
| Malic acid | 917.03 (377.31, 1756.33) | 939.49 (472.75, 1577.93) | 0.059206 | 0.899 | 0.08 |
| Malonic acid | 311.87 (196.85, 447.21) | 227.20 (115.92, 296.25) | 0.142491 | 0.023 | 0.011 |
| Melibiose | 116.18 (50.39, 217.20) | 38.03 (32.21, 97.20) | 0.138663 | 0.002 | 0.018 |
| Methylmalonic acid | 818.91 (446.01, 1074.32) | 569.72 (334.67, 892.23) | 0.123103 | 0.106 | 0.008 |
| Mevalonic acid | 4947.15 (2678.15, 9295.74) | 3295.37 (2241.32, 5173.77) | 0.680908 | 0.049 | 0.055 |
| N-acetyl-d-glucosamine | 4912.35 (3377.44, 7766.58) | 2620.09 (1577.94, 4571.43) | 1.033435 | <.001 | 0.006 |
| Nicotinic acid | 29.06 (12.07, 54.67) | 7.98 (3.05, 20.05) | 0.082852 | <.001 | 0.024 |
| Oxalic acid | 12053.45 (7366.81, 16898.59) | 9290.59 (7703.49, 15349.32) | 0.65011 | 0.847 | 0.285 |
| Pantothenic acid | 4204.93 (2326.60, 7470.67) | 2658.83 (1551.10, 4484.59) | 0.659458 | 0.045 | 0.01 |
| Phosphoryl choline | 347.06 (230.85, 608.88) | 271.02 (100.08, 482.32) | 0.117834 | 0.246 | 0.031 |
| Pyruvic acid | 6621.42 (4582.48, 11799.59) | 6478.87 (4291.13, 12638.11) | 0.060732 | 0.804 | 0.164 |
| Ribose 5-phosphate | 1147.09 (857.79, 1880.93) | 1470.37 (1058.49, 1928.02) | 0.058938 | 0.081 | 0.785 |
| Succinic acid | 4306.48 (2759.14, 9084.47) | 3404.96 (2145.37, 4421.33) | 0.641227 | 0.021 | 0.017 |
| Trehalose 6-phosphate | 0.24 (0.00, 3.27) | 0.56 (0.00, 2.83) | 5.16E-05 | 0.718 | 0.129 |
| Uridine 5'-monophosphate | 11.08 (7.33, 16.54) | 9.64 (4.61, 15.12) | 0.022662 | 0.208 | 0.047 |
| Xylose | 61017.59 (38330.48, 88428.20) | 31602.82 (20597.82, 40490.59) | 3.465209 | <.001 | 0.001 |
| UC and CG (control) represent the distribution of metabolite quantitative data for the two groups, presented as median values with interquartile ranges (25th and 75th percentiles). For metabolite levels, statistics was performed using t test for normal distribution, using Mann–Whitney test for non-normal distribution. VIP was obtained from OPLS-DA model with a threshold of 1.0. Padj values from multiple logistic regression analysis with disease status as the dependent variable, metabolites as the independent variable, and age, sex, smoking, weight and height as covariates. Padj <0.05 in bold. | | | | | |

Table S2. Metabolite differences between CD and control group (CG).

| Metabolite | CD | CG | VIP | P | Padj |
| --- | --- | --- | --- | --- | --- |
| 2-deoxy-d-glucose | 1573.36 (958.42, 2757.42) | 1417.06 (800.53, 1946.46) | 0.156404489 | 0.373 | 0.016 |
| 2-isopropylmalic acid | 55.09 (26.67, 147.98) | 13.60 (11.51, 23.76) | 0.149123298 | 0.003 | 0.196 |
| 2-ketoglutaric acid | 9350.43 (4101.76, 14396.95) | 7616.52 (4249.13, 12536.18) | 0.347674228 | 0.606 | 0.383 |
| 3-aminoisobutanoic acid | 44900.95 (11546.44, 96076.50) | 5314.07 (1657.85, 24138.93) | 1.44003881 | 0.006 | 0.092 |
| 3-phosphoglyceric acid | 1160.88 (413.14, 2202.17) | 706.08 (520.41, 1196.12) | 0.163507492 | 0.189 | 0.393 |
| 5'-guanylic acid | 4.69 (2.57, 7.44) | 4.23 (3.03, 7.50) | 0.002890781 | 0.852 | 0.785 |
| Adenosine 5'-monophosphate | 93.72 (68.10, 117.05) | 133.39 (75.72, 212.53) | 0.05267483 | 0.047 | 0.075 |
| Cis-aconitic acid | 44740.09 (35477.26, 82194.37) | 44453.06 (26554.47, 60185.30) | 1.488169824 | 0.339 | 0.073 |
| Citric acid | 98604.86 (64819.40, 165294.85) | 232073.59 (131585.43, 332350.03) | 3.864678577 | 0.004 | 0.06 |
| Ethylmalonic acid | 1730.43 (1058.09, 2886.11) | 1237.87 (636.57, 1639.62) | 0.333654848 | 0.052 | 0.04 |
| Fructose | 4485.14 (3265.46, 7099.33) | 2323.17 (1374.36, 6034.46) | 0.254562085 | 0.067 | 0.131 |
| Fructose 6-phosphate | 157.78 (107.06, 256.22) | 163.78 (111.71, 237.74) | 0.002325982 | 0.546 | 0.501 |
| Fumaric acid | 307.18 (137.59, 466.28) | 332.69 (167.98, 604.26) | 0.036416565 | 0.503 | 0.678 |
| Galactose | 4970.27 (2495.29, 6964.31) | 3310.66 (2403.64, 5527.36) | 0.035055791 | 0.23 | 0.633 |
| Galactose 1-phosphate | 106.82 ± 61.39 | 83.67 ± 42.06 | 0.043338826 | 0.158 | 0.146 |
| Glucaric acid | 3628.63 (2289.84, 8390.62) | 2675.87 (1744.53, 4107.68) | 0.639503337 | 0.058 | 0.013 |
| Gluconic acid | 82367.98 (62535.14, 115881.95) | 152736.32 (57067.21, 358477.58) | 4.095667822 | 0.052 | 0.157 |
| Glucosamine 6-phosphate | 11.48 (10.87, 12.30) | 11.76 (10.91, 14.85) | 0.008986573 | 0.462 | 0.543 |
| Glucose | 11334.07 (8878.48, 22241.77) | 19736.87 (9644.03, 32298.40) | 1.658663504 | 0.221 | 0.19 |
| Glucose 1-phosphate | 53.39 (7.94, 134.86) | 100.41 (44.56, 329.82) | 0.112690396 | 0.088 | 0.682 |
| Glucose 6-phosphate | 202.06 (92.96, 512.19) | 223.04 (133.25, 380.03) | 0.085878412 | 0.818 | 0.638 |
| Glyceraldehyde | 308.05 (231.82, 437.17) | 225.12 (167.44, 330.67) | 0.109199115 | 0.049 | 0.044 |
| Glyceric acid | 1587.29 (1339.61, 2864.13) | 1267.49 (820.31, 2133.33) | 0.1984442 | 0.339 | 0.496 |
| Glycolic acid | 20228.14 ± 12166.96 | 14662.44 ± 6250.41 | 0.879137472 | 0.073 | 0.019 |
| Glyoxylic acid | 384.13 ± 235.39 | 327.11 ± 150.07 | 0.095345657 | 0.323 | 0.051 |
| Isocitric acid | 24013.39 (15634.41, 38688.46) | 16146.56 (12225.60, 21714.61) | 0.922650365 | 0.064 | 0.014 |
| Isonicotinic acid | 1.09 (0.57, 2.11) | 0.72 (0.30, 1.06) | 0.008787634 | 0.13 | 0.151 |
| Itaconic acid | 197.91 (123.46, 460.69) | 199.95 (126.45, 280.79) | 0.09677781 | 0.75 | 0.195 |
| Lactose | 5465.53 (3020.34, 8471.26) | 6530.46 (2506.34, 16316.06) | 0.523951286 | 0.621 | 0.29 |
| L-carnitine | 11063.82 (2312.78, 36155.60) | 10824.00 (6497.61, 58137.20) | 0.009503547 | 0.561 | 0.845 |
| L-fucose | 22525.34 (8854.73, 27738.97) | 8578.86 (6501.16, 13691.53) | 1.141766031 | 0.006 | 0.011 |
| Lipoic acid | 5.11 (2.41, 7.89) | 5.12 (2.03, 7.28) | 0.00867624 | 0.591 | 0.689 |
| L-rhamnose | 406.38 (236.46, 647.18) | 326.12 (213.24, 380.73) | 0.1055577 | 0.205 | 0.044 |
| Malic acid | 626.25 (349.90, 1178.38) | 939.49 (472.75, 1577.93) | 0.105359667 | 0.182 | 0.312 |
| Malonic acid | 357.55 ± 235.39 | 236.18 ± 140.12 | 0.102956103 | 0.035 | 0.065 |
| Melibiose | 42.36 (26.02, 93.47) | 38.03 (32.21, 97.20) | 0.011192618 | 0.767 | 0.333 |
| Methylmalonic acid | 773.58 (542.31, 1058.32) | 569.72 (334.67, 892.23) | 0.101447757 | 0.114 | 0.016 |
| Mevalonic acid | 3965.56 (2161.26, 9392.03) | 3295.37 (2241.32, 5173.77) | 0.492291043 | 0.306 | 0.08 |
| N-acetyl-d-glucosamine | 6024.71 (3091.22, 9866.60) | 2620.09 (1577.94, 4571.43) | 0.606737236 | 0.003 | 0.01 |
| Nicotinic acid | 46.63 (14.88, 122.37) | 7.98 (3.05, 20.05) | 0.085431724 | <.001 | 0.039 |
| Oxalic acid | 9698.60 (5390.75, 13157.53) | 9290.59 (7703.49, 15349.32) | 0.070704894 | 0.35 | 0.227 |
| Pantothenic acid | 5835.06 (2004.72, 9090.89) | 2658.83 (1551.10, 4484.59) | 0.493137605 | 0.032 | 0.021 |
| Phosphoryl choline | 242.37 (124.09, 659.32) | 271.02 (100.08, 482.32) | 0.115460275 | 0.701 | 0.366 |
| Pyruvic acid | 9663.97 (6746.79, 19776.59) | 6478.87 (4291.13, 12638.11) | 0.455767612 | 0.142 | 0.094 |
| Ribose 5-phosphate | 1293.61 (921.83, 1553.65) | 1470.37 (1058.49, 1928.02) | 0.040539471 | 0.296 | 0.501 |
| Succinic acid | 4272.69 (2507.76, 7172.37) | 3404.96 (2145.37, 4421.33) | 0.369286373 | 0.213 | 0.844 |
| Trehalose 6-phosphate | 0.40 (0.00, 2.92) | 0.56 (0.00, 2.83) | 0.022657565 | 0.973 | 0.577 |
| Uridine 5'-monophosphate | 8.30 (3.83, 12.63) | 9.64 (4.61, 15.12) | 0.003010752 | 0.503 | 0.613 |
| Xylose | 74032.76 (29155.39, 108155.68) | 31602.82 (20597.82, 40490.59) | 2.214117509 | 0.002 | 0.007 |
| CD and CG (control) represent the distribution of metabolite quantitative data for the two groups, presented as median values with interquartile ranges (25th and 75th percentiles). For metabolite levels, statistics was performed using t test for normal distribution, using Mann–Whitney test for non-normal distribution. VIP was obtained from OPLS-DA model with a threshold of 1.0. Padj values from multiple logistic regression analysis with disease status as the dependent variable, metabolites as the independent variable, and age, sex, smoking, weight and height as covariates. Padj <0.05 in bold. | | | | | |
