# Supplementary material for: Targeted urinary metabolomics combined with machine learning to identify biomarkers related to central carbon metabolism for IBD
Source: Front Mol Biosci. 2025 Aug 11;12:1615047. doi: 10.3389/fmolb.2025.1615047 (PMC12375463; doi:10.3389/fmolb.2025.1615047)
Supplement: Supplementary file 2 [file Table2.docx]

# Table S3. Ion Pair Information of Metabolites

| The ion pair information of metabolites | | | | |
| --- | --- | --- | --- | --- |
| Metabolite | Parent ion | Fragment Ion | Declustering Potential | Collision Energy |
| L-Carnitine | 297 | 238.3 | 45 | 30 |
| Phosphoryl choline | 319.3 | 166 | 65 | 27 |
| 5'-Guanylic acid | 499.2 | 152 | 70 | 24 |
| Glucose | 314.2 | 206 | -60 | -17 |
| Glyceric acid | 240 | 137 | -45 | -24 |
| Galactose l-phosphate | 394 | 79.1 | -35 | -80 |
| Lactose | 476.1 | 179.2 | -60 | -20 |
| Xylose | 284.2 | 206 | -45 | -14 |
| Galactose | 314.2 | 206.1 | -60 | -17 |
| L-Rhamnose | 298.1 | 206.1 | -55 | -13 |
| Nicotinic acid | 257.1 | 150.1 | -90 | -27 |
| Glyceraldehyde | 223.9 | 205.9 | -40 | -11 |
| Gluconic acid | 330 | 210 | -40 | -25 |
| Itaconic acid | 399.2 | 246 | -40 | -27 |
| cis-Aconitic acid | 578.3 | 425.2 | -50 | -23 |
| Isocitric acid | 596.3 | 387.3 | -50 | -24 |
| Succinic acid | 387.1 | 234.2 | -100 | -24 |
| Malic acid | 403 | 208.1 | -85 | -25 |
| Adenosine 5'-monophosphate | 481.1 | 79 | -40 | -100 |
| 3-Phosphoglyceric acid | 455.1 | 232.1 | -40 | -27 |
| 2-Ketoglutaric acid | 550.1 | 232.9 | -100 | -37 |
| Glucose l-phosphate | 394.1 | 232.2 | -40 | -26 |
| 2-Deoxy-D-glucose | 298.1 | 219.8 | -45 | -13 |
| Fructose | 314.2 | 236 | -50 | -14 |
| Melibiose | 476.1 | 206.2 | -40 | -25 |
| L-Fucose | 298.1 | 206 | -55 | -13 |
| Glucosamine 6-phosphate | 393.1 | 232 | -35 | -28 |
| Fumaric acid | 385.1 | 232.1 | -120 | -25 |
| Fructose 6-phosphate | 529 | 232.1 | -40 | -32 |
| Glucose 6-phosphate | 529 | 232 | -40 | -32 |
| Pyruvic acid | 357 | 137.1 | -100 | -27 |
| Citric acid | 596.3 | 137 | -35 | -66 |
| Glyoxylic acid | 343.2 | 178 | -25 | -23 |
| N-acetyl-D-glucosamine | 355.1 | 235.2 | -30 | -18 |
| Pantothenic acid | 353 | 206.1 | -55 | -27 |
| Uridine 5'-monophosphate | 458.1 | 78.9 | -55 | -95 |
| Ribose 5-phosphate | 499.1 | 232.3 | -50 | -30 |
| Glucaric acid | 479.1 | 208.3 | -130 | -36 |
| Mevalonic acid | 282.1 | 194 | -70 | -20 |
| Trehalose 6-phosphate | 556.1 | 376.1 | -50 | -43 |
| Glycolic acid | 210 | 137.1 | -50 | -22 |
| Isonicotinic acid | 257.1 | 150 | -90 | -27 |
| Ethylmalonic acid | 401 | 178.2 | -120 | -27 |
| Oxalic acid | 359.2 | 150.1 | -130 | -29 |
| Lipoic acid | 340.1 | 306.1 | -125 | -17 |
| 2-Isopropylmalic acid | 445.2 | 250 | -90 | -27 |
| 3-Aminoisobutanoic acid | 236.9 | 136.8 | -110 | -27 |
| Malonic acid | 373 | 177.9 | -90 | -23 |
| Methylmalonic acid | 387 | 177.8 | -100 | -25 |
