# Supplementary material for: Targeted urinary metabolomics combined with machine learning to identify biomarkers related to central carbon metabolism for IBD
Source: Front Mol Biosci. 2025 Aug 11;12:1615047. doi: 10.3389/fmolb.2025.1615047 (PMC12375463; doi:10.3389/fmolb.2025.1615047)
Supplement: Supplementary file 3 [file DataSheet1.pdf]

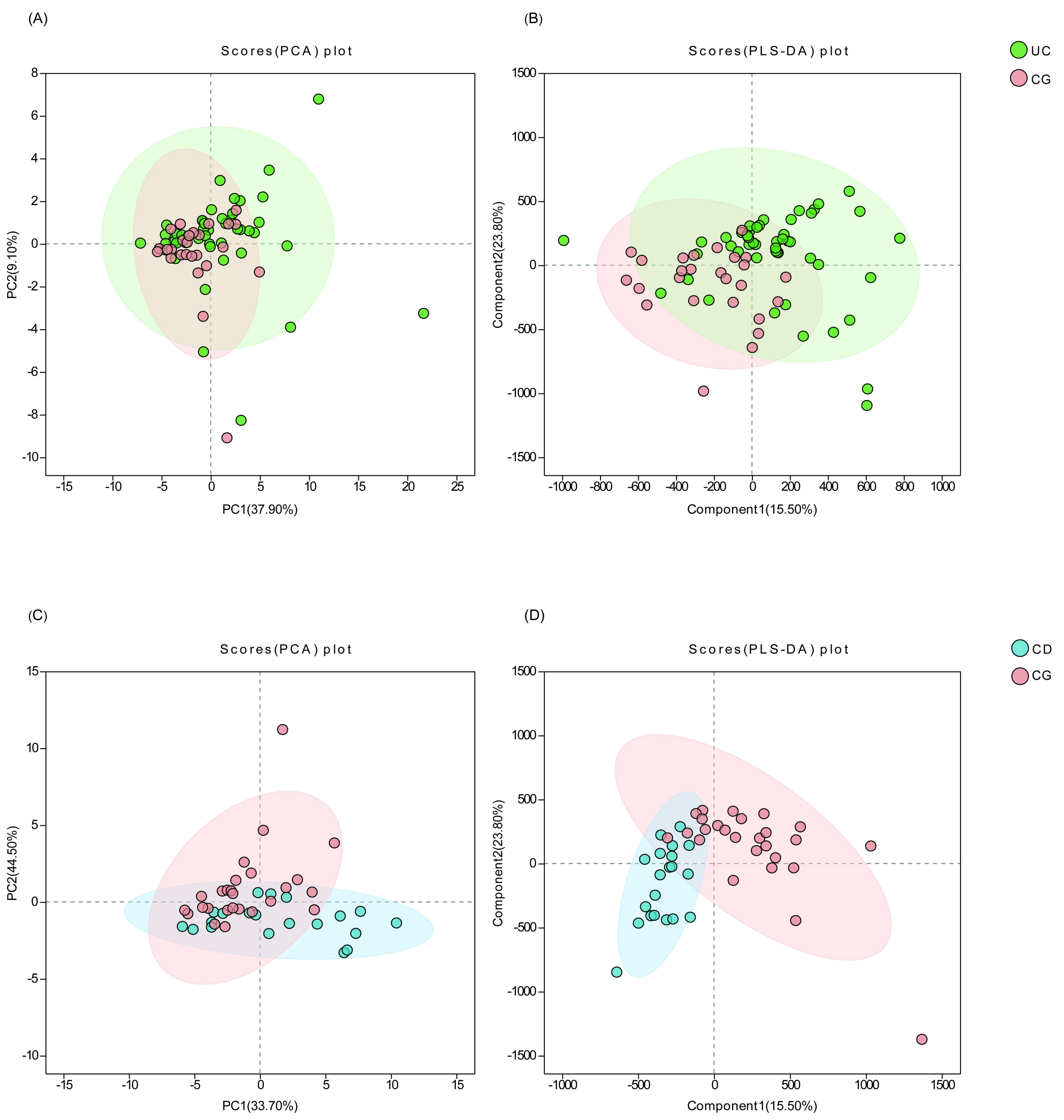

Supplementary Fig S1 Multivariate statistical analysis: PCA and PLS-DA.

(A) PCA score plot of UC (green dots) vs CG (pink dots); (B) PLS-DA score plot of UC (green dots) vs CG (pink dots); (C) PCA score plot of CD (blue dots) vs CG (pink dots); (D) PLS-DA score plot of CD (blue dots) vs CG (pink dots).
