# Supplementary material for: Targeted urinary metabolomics combined with machine learning to identify biomarkers related to central carbon metabolism for IBD
Source: Front Mol Biosci. 2025 Aug 11;12:1615047. doi: 10.3389/fmolb.2025.1615047 (PMC12375463; doi:10.3389/fmolb.2025.1615047)
Supplement: Supplementary file 4 [file Table3.docx]

TableS4. Precision and Recovery of Central Carbon Metabolites

| Analyte | Concentration (ng/mL) | Intra-day Precision (%) (n=6) | Inter-day Precision (%) (n=18) | Recovery (%) (n=6) |
| --- | --- | --- | --- | --- |
| L-Carnitine | 100 | 2.08 | 2.72 | 102.23 ± 2.13 |
| L-Carnitine | 500 | 2.42 | 2.55 | 99.93 ± 2.42 |
| L-Carnitine | 1600 | 2.34 | 2.33 | 96.10 ± 2.24 |
| Phosphoryl choline | 100 | 3.32 | 7.78 | 99.02 ± 3.29 |
| Phosphoryl choline | 500 | 2.2 | 3.13 | 98.23 ± 2.16 |
| Phosphoryl choline | 1600 | 4.7 | 6.85 | 102.08 ± 4.80 |
| 5'-Guanylic acid | 25 | 7.27 | 8.81 | 93.08 ± 6.77 |
| 5'-Guanylic acid | 125 | 7.46 | 8.09 | 89.44 ± 6.67 |
| 5'-Guanylic acid | 400 | 4.42 | 10.6 | 85.33 ± 3.77 |
| Glucose | 2500 | 4.06 | 5.66 | 109.74 ± 4.45 |
| Glucose | 12500 | 5.67 | 4.68 | 113.31 ± 6.42 |
| Glucose | 40000 | 4.81 | 4.43 | 99.96 ± 4.81 |
| Glyceric acid | 100 | 3.73 | 3.14 | 99.11 ± 3.70 |
| Glyceric acid | 500 | 1.92 | 3.2 | 103.20 ± 1.99 |
| Glyceric acid | 1600 | 2.0 | 3.12 | 105.78 ± 2.12 |
| Galactose 1-phosphate | 100 | 7.53 | 9.9 | 101.00 ± 7.61 |
| Galactose 1-phosphate | 500 | 5.23 | 5.88 | 97.64 ± 5.10 |
| Galactose 1-phosphate | 1600 | 4.43 | 10.71 | 90.86 ± 4.03 |
| Lactose | 250 | 4.9 | 9.91 | 97.79 ± 4.79 |
| Lactose | 1250 | 9.24 | 9.09 | 105.26 ± 9.73 |
| Lactose | 4000 | 10.06 | 10.97 | 103.48 ± 10.41 |
| Xylose | 2500 | 5.4 | 6.69 | 110.57 ± 5.97 |
| Xylose | 12500 | 9.3 | 9.41 | 106.88 ± 9.94 |
| Xylose | 40000 | 6.7 | 6.83 | 108.54 ± 7.27 |
| Galactose | 2500 | 4.33 | 4.95 | 101.84 ± 4.41 |
| Galactose | 12500 | 3.21 | 5.13 | 108.33 ± 3.48 |
| Galactose | 40000 | 2.2 | 4.12 | 112.43 ± 2.47 |
| L-Rhamnose | 200 | 8.91 | 8.15 | 106.57 ± 9.49 |
| L-Rhamnose | 1000 | 6.0 | 10.09 | 105.65 ± 6.34 |
| L-Rhamnose | 3200 | 9.23 | 11.07 | 99.75 ± 9.21 |
| Nicotinic acid | 100 | 3.25 | 3.57 | 103.51 ± 3.36 |
| Nicotinic acid | 500 | 2.58 | 2.43 | 99.43 ± 2.56 |
| Nicotinic acid | 1600 | 3.57 | 5.1 | 92.19 ± 3.30 |
| Glyceraldehyde | 50 | 1.64 | 3.06 | 105.83 ± 1.74 |
| Glyceraldehyde | 250 | 2.28 | 2.95 | 102.12 ± 2.33 |
| Glyceraldehyde | 800 | 3.78 | 5.32 | 94.36 ± 3.57 |
| Gluconic acid | 250 | 4.33 | 6.11 | 111.73 ± 4.83 |
| Gluconic acid | 1250 | 2.05 | 3.4 | 110.74 ± 2.27 |
| Gluconic acid | 4000 | 2.92 | 7.79 | 100.30 ± 2.93 |
| Itaconic acid | 25 | 3.06 | 3.17 | 104.51 ± 3.20 |
| Itaconic acid | 125 | 3.15 | 4.55 | 104.54 ± 3.29 |
| Itaconic acid | 400 | 3.4 | 3.94 | 94.12 ± 3.20 |
| cis-Aconitic acid | 100 | 3.76 | 4.48 | 113.37 ± 4.26 |
| cis-Aconitic acid | 500 | 3.81 | 3.77 | 110.38 ± 4.21 |
| cis-Aconitic acid | 1600 | 3.63 | 3.64 | 107.93 ± 3.92 |
| Isocitric acid | 100 | 5.93 | 8.1 | 101.00 ± 5.99 |
| Isocitric acid | 500 | 3.77 | 7.14 | 99.01 ± 3.73 |
| Isocitric acid | 1600 | 4.98 | 5.58 | 91.42 ± 4.55 |
| Succinic acid | 100 | 2.93 | 4.14 | 102.50 ± 3.01 |
| Succinic acid | 500 | 4.08 | 4.21 | 109.89 ± 4.48 |
| Succinic acid | 1600 | 4.22 | 3.74 | 105.21 ± 4.44 |
| Malic acid | 100 | 5.56 | 3.94 | 99.62 ± 5.54 |
| Malic acid | 500 | 2.01 | 6.38 | 101.69 ± 2.04 |
| Malic acid | 1600 | 1.2 | 3.81 | 100.55 ± 1.21 |
| Adenosine 5'-monophosphate | 50 | 2.74 | 4.83 | 98.11 ± 2.69 |
| Adenosine 5'-monophosphate | 250 | 2.4 | 4.28 | 96.29 ± 2.31 |
| Adenosine 5'-monophosphate | 800 | 3.4 | 7.61 | 91.45 ± 3.11 |
| 3-Phosphoglyceric acid | 100 | 4.92 | 4.19 | 101.38 ± 4.99 |
| 3-Phosphoglyceric acid | 500 | 3.03 | 3.05 | 100.74 ± 3.05 |
| 3-Phosphoglyceric acid | 1600 | 3.77 | 7.43 | 93.79 ± 3.54 |
| 2-Ketoglutaric acid | 100 | 2.65 | 4.32 | 98.30 ± 2.61 |
| 2-Ketoglutaric acid | 500 | 2.95 | 4.14 | 101.65 ± 3.00 |
| 2-Ketoglutaric acid | 1600 | 2.39 | 3.83 | 102.20 ± 2.44 |
| Glucose 1-phosphate | 100 | 9.32 | 10.15 | 97.63 ± 9.09 |
| Glucose 1-phosphate | 500 | 2.93 | 6.02 | 92.29 ± 2.70 |
| Glucose 1-phosphate | 1600 | 5.8 | 10.94 | 86.57 ± 5.02 |
| 2-Deoxy-D-glucose | 100 | 8.56 | 12.66 | 106.45 ± 9.11 |
| 2-Deoxy-D-glucose | 500 | 6.67 | 11.93 | 110.35 ± 7.36 |
| 2-Deoxy-D-glucose | 1600 | 8.35 | 8.84 | 100.97 ± 8.43 |
| Fructose | 100 | 11.92 | 9.89 | 107.45 ± 12.81 |
| Fructose | 500 | 7.18 | 6.53 | 111.53 ± 8.01 |
| Fructose | 1600 | 11.15 | 8.62 | 104.16 ± 11.62 |
| Melibiose | 200 | 5.46 | 9.18 | 102.02 ± 5.58 |
| Melibiose | 1000 | 8.22 | 8.84 | 94.77 ± 7.79 |
| Melibiose | 3200 | 8.13 | 8.91 | 94.93 ± 7.71 |
| L-Fucose | 100 | 8.76 | 7.08 | 98.93 ± 8.66 |
| L-Fucose | 500 | 9.17 | 7.73 | 105.86 ± 9.71 |
| L-Fucose | 1600 | 2.43 | 8.29 | 98.57 ± 2.40 |
| Glucosamine 6-phosphate | 100 | 3.16 | 4.27 | 100.25 ± 3.17 |
| Glucosamine 6-phosphate | 500 | 3.42 | 4.04 | 97.32 ± 3.33 |
| Glucosamine 6-phosphate | 1600 | 3.16 | 7.75 | 88.74 ± 2.81 |
| Fumaric acid | 50 | 2.47 | 3.15 | 103.72 ± 2.56 |
| Fumaric acid | 250 | 2.36 | 4.12 | 101.81 ± 2.40 |
| Fumaric acid | 800 | 2.96 | 3.46 | 93.22 ± 2.76 |
| Fructose 6-phosphate | 100 | 7.15 | 7.93 | 105.34 ± 7.53 |
| Fructose 6-phosphate | 500 | 5.15 | 8.47 | 96.64 ± 4.97 |
| Fructose 6-phosphate | 1600 | 3.67 | 10.32 | 86.05 ± 3.16 |
| Glucose 6-phosphate | 50 | 4.64 | 4.75 | 106.74 ± 4.95 |
| Glucose 6-phosphate | 250 | 2.52 | 6.57 | 101.49 ± 2.55 |
| Glucose 6-phosphate | 800 | 1.92 | 6.83 | 96.36 ± 1.85 |
| Pyruvic acid | 100 | 4.2 | 4.88 | 102.36 ± 4.29 |
| Pyruvic acid | 500 | 1.78 | 4.91 | 104.32 ± 1.85 |
| Pyruvic acid | 1600 | 2.0 | 3.64 | 105.66 ± 2.11 |
| Citric acid | 2500 | 4.14 | 4.87 | 100.71 ± 4.17 |
| Citric acid | 12500 | 3.38 | 8.84 | 106.76 ± 3.60 |
| Citric acid | 40000 | 2.4 | 4.93 | 107.73 ± 2.59 |
| Glyoxylic acid | 100 | 3.23 | 3.94 | 99.03 ± 3.19 |
| Glyoxylic acid | 500 | 3.37 | 3.81 | 97.48 ± 3.29 |
| Glyoxylic acid | 1600 | 3.15 | 5.1 | 96.04 ± 3.02 |
| N-acetyl-D-glucosamine | 250 | 4.99 | 8.55 | 113.71 ± 5.68 |
| N-acetyl-D-glucosamine | 1250 | 6.27 | 6.96 | 108.24 ± 6.79 |
| N-acetyl-D-glucosamine | 4000 | 3.94 | 7.59 | 103.13 ± 4.06 |
| Pantothenic acid | 100 | 4.52 | 3.73 | 95.81 ± 4.33 |
| Pantothenic acid | 500 | 2.21 | 3.62 | 98.68 ± 2.18 |
| Pantothenic acid | 1600 | 2.09 | 2.83 | 108.28 ± 2.26 |
| Uridine 5'-monophosphate | 25 | 4.07 | 3.91 | 107.17 ± 4.36 |
| Uridine 5'-monophosphate | 125 | 3.41 | 4.38 | 100.46 ± 3.43 |
| Uridine 5'-monophosphate | 400 | 6.13 | 9.04 | 90.97 ± 5.58 |
| Ribose 5-phosphate | 100 | 13.19 | 12.87 | 89.38 ± 11.79 |
| Ribose 5-phosphate | 500 | 6.85 | 8.07 | 98.97 ± 6.78 |
| Ribose 5-phosphate | 1600 | 4.4 | 8.9 | 91.19 ± 4.01 |
| Glucaric acid | 100 | 7.2 | 5.83 | 110.12 ± 7.93 |
| Glucaric acid | 500 | 3.05 | 4.52 | 102.99 ± 3.14 |
| Glucaric acid | 1600 | 3.78 | 6.63 | 96.49 ± 3.65 |
| Mevalonic acid | 100 | 3.75 | 4.09 | 102.46 ± 3.84 |
| Mevalonic acid | 500 | 4.31 | 3.9 | 100.07 ± 4.32 |
| Mevalonic acid | 1600 | 4.95 | 5.17 | 94.58 ± 4.68 |
| Trehalose 6-phosphate | 25 | 6.01 | 6.71 | 103.94 ± 6.24 |
| Trehalose 6-phosphate | 125 | 2.99 | 3.61 | 101.47 ± 3.04 |
| Trehalose 6-phosphate | 400 | 5.23 | 6.26 | 95.13 ± 4.97 |
| Glycolic acid | 100 | 2.81 | 4.92 | 98.96 ± 2.78 |
| Glycolic acid | 500 | 3.78 | 4.98 | 104.79 ± 3.96 |
| Glycolic acid | 1600 | 2.83 | 4.6 | 103.53 ± 2.93 |
| Isonicotinic acid | 25 | 5.01 | 4.43 | 98.81 ± 4.95 |
| Isonicotinic acid | 125 | 3.05 | 4.26 | 97.94 ± 2.99 |
| Isonicotinic acid | 400 | 4.09 | 6.09 | 91.97 ± 3.76 |
| Ethylmalonic acid | 50 | 2.82 | 5.04 | 104.37 ± 2.94 |
| Ethylmalonic acid | 250 | 2.69 | 3.57 | 99.30 ± 2.67 |
| Ethylmalonic acid | 800 | 2.05 | 6.0 | 106.30 ± 2.18 |
| Oxalic acid | 250 | 4.4 | 5.16 | 99.75 ± 4.39 |
| Oxalic acid | 1250 | 1.79 | 3.73 | 104.94 ± 1.87 |
| Oxalic acid | 4000 | 1.97 | 4.03 | 112.75 ± 2.22 |
| Lipoic acid | 25 | 3.3 | 4.08 | 98.42 ± 3.25 |
| Lipoic acid | 125 | 3.81 | 3.93 | 97.35 ± 3.70 |
| Lipoic acid | 400 | 4.11 | 4.0 | 88.59 ± 3.64 |
| 2-Isopropylmalic acid | 25 | 2.33 | 4.53 | 96.42 ± 2.25 |
| 2-Isopropylmalic acid | 125 | 2.43 | 3.08 | 104.05 ± 2.53 |
| 2-Isopropylmalic acid | 400 | 3.06 | 2.31 | 106.67 ± 3.26 |
| 3-Aminoisobutanoic acid | 50 | 5.99 | 5.06 | 97.88 ± 5.86 |
| 3-Aminoisobutanoic acid | 250 | 2.08 | 5.61 | 102.43 ± 2.13 |
| 3-Aminoisobutanoic acid | 800 | 1.72 | 3.2 | 106.81 ± 1.83 |
| Malonic acid | 50 | 4.79 | 5.21 | 98.71 ± 4.73 |
| Malonic acid | 250 | 2.0 | 2.66 | 105.40 ± 2.10 |
| Malonic acid | 800 | 3.97 | 4.25 | 110.66 ± 4.40 |
| Methylmalonic acid | 25 | 1.62 | 7.15 | 107.80 ± 1.75 |
| Methylmalonic acid | 125 | 3.95 | 4.03 | 96.74 ± 3.82 |
| Methylmalonic acid | 400 | 4.21 | 6.4 | 96.81 ± 4.08 |
