# Supplementary material for: Targeted urinary metabolomics combined with machine learning to identify biomarkers related to central carbon metabolism for IBD
Source: Front Mol Biosci. 2025 Aug 11;12:1615047. doi: 10.3389/fmolb.2025.1615047 (PMC12375463; doi:10.3389/fmolb.2025.1615047)
Supplement: Supplementary file 5 [file Table4.docx]

# Table 5.1 Standard Concentrations for Calibration Curves of Central Carbon Metabolites

| Analyte | Concentration (ng/ml) | | | | | | | | | | | |
| --- | --- | --- | --- | --- | --- | --- | --- | --- | --- | --- | --- | --- |
|  | Level 1 | Level 2 | Level 3 | Level 4 | Level 5 | Level 6 | Level 7 | Level 8 | Level 9 | Level 10 | Level 11 | Level 12 |
| L-Carnitine | 0.5 | 1 | 2 | 5 | 10 | 20 | 50 | 100 | 200 | 500 | 1000 | 2000 |
| Phosphoryl choline | 0.5 | 1 | 2 | 5 | 10 | 20 | 50 | 100 | 200 | 500 | 1000 | 2000 |
| 5'-Guanylic acid | 0.125 | 0.25 | 0.5 | 1.25 | 2.5 | 5 | 12.5 | 25 | 50 | 125 | 250 | 500 |
| Glucose | 12.5 | 25 | 50 | 125 | 250 | 500 | 1250 | 2500 | 5000 | 12500 | 25000 | 50000 |
| Glyceric acid | 0.5 | 1 | 2 | 5 | 10 | 20 | 50 | 100 | 200 | 500 | 1000 | 2000 |
| Galactose 1-phosphate | 0.5 | 1 | 2 | 5 | 10 | 20 | 50 | 100 | 200 | 500 | 1000 | 2000 |
| Lactose | 1.25 | 2.5 | 5 | 12.5 | 25 | 50 | 125 | 250 | 500 | 1250 | 2500 | 5000 |
| Xylose | 12.5 | 25 | 50 | 125 | 250 | 500 | 1250 | 2500 | 5000 | 12500 | 25000 | 50000 |
| Galactose | 12.5 | 25 | 50 | 125 | 250 | 500 | 1250 | 2500 | 5000 | 12500 | 25000 | 50000 |
| L-Rhamnose | 1 | 2 | 4 | 10 | 20 | 40 | 100 | 200 | 400 | 1000 | 2000 | 4000 |
| Nicotinic acid | 0.5 | 1 | 2 | 5 | 10 | 20 | 50 | 100 | 200 | 500 | 1000 | 2000 |
| Glyceraldehyde | 0.25 | 0.5 | 1 | 2.5 | 5 | 10 | 25 | 50 | 100 | 250 | 500 | 1000 |
| Gluconic acid | 1.25 | 2.5 | 5 | 12.5 | 25 | 50 | 125 | 250 | 500 | 1250 | 2500 | 5000 |
| Itaconic acid | 0.125 | 0.25 | 0.5 | 1.25 | 2.5 | 5 | 12.5 | 25 | 50 | 125 | 250 | 500 |
| cis-Aconitic acid | 0.5 | 1 | 2 | 5 | 10 | 20 | 50 | 100 | 200 | 500 | 1000 | 2000 |
| Isocitric acid | 0.5 | 1 | 2 | 5 | 10 | 20 | 50 | 100 | 200 | 500 | 1000 | 2000 |
| Succinic acid | 0.5 | 1 | 2 | 5 | 10 | 20 | 50 | 100 | 200 | 500 | 1000 | 2000 |
| Malic acid | 0.5 | 1 | 2 | 5 | 10 | 20 | 50 | 100 | 200 | 500 | 1000 | 2000 |
| Adenosine 5'-monophosphate | 0.25 | 0.5 | 1 | 2.5 | 5 | 10 | 25 | 50 | 100 | 250 | 500 | 1000 |
| 3-Phosphoglyceric acid | 0.5 | 1 | 2 | 5 | 10 | 20 | 50 | 100 | 200 | 500 | 1000 | 2000 |
| 2-Ketoglutaric acid | 0.5 | 1 | 2 | 5 | 10 | 20 | 50 | 100 | 200 | 500 | 1000 | 2000 |
| Glucose 1-phosphate | 0.5 | 1 | 2 | 5 | 10 | 20 | 50 | 100 | 200 | 500 | 1000 | 2000 |
| 2-Deoxy-D-glucose | 0.5 | 1 | 2 | 5 | 10 | 20 | 50 | 100 | 200 | 500 | 1000 | 2000 |
| Fructose | 0.5 | 1 | 2 | 5 | 10 | 20 | 50 | 100 | 200 | 500 | 1000 | 2000 |
| Melibiose | 1 | 2 | 4 | 10 | 20 | 40 | 100 | 200 | 400 | 1000 | 2000 | 4000 |
| L-Fucose | 0.5 | 1 | 2 | 5 | 10 | 20 | 50 | 100 | 200 | 500 | 1000 | 2000 |
| Glucosamine 6-phosphate | 0.5 | 1 | 2 | 5 | 10 | 20 | 50 | 100 | 200 | 500 | 1000 | 2000 |
| Fumaric acid | 0.25 | 0.5 | 1 | 2.5 | 5 | 10 | 25 | 50 | 100 | 250 | 500 | 1000 |
| Fructose 6-phosphate | 0.5 | 1 | 2 | 5 | 10 | 20 | 50 | 100 | 200 | 500 | 1000 | 2000 |
| Glucose 6-phosphate | 0.25 | 0.5 | 1 | 2.5 | 5 | 10 | 25 | 50 | 100 | 250 | 500 | 1000 |
| Pyruvic acid | 0.5 | 1 | 2 | 5 | 10 | 20 | 50 | 100 | 200 | 500 | 1000 | 2000 |
| Citric acid | 12.5 | 25 | 50 | 125 | 250 | 500 | 1250 | 2500 | 5000 | 12500 | 25000 | 50000 |
| Glyoxylic acid | 0.5 | 1 | 2 | 5 | 10 | 20 | 50 | 100 | 200 | 500 | 1000 | 2000 |
| N-acetyl-D-glucosamine | 1.25 | 2.5 | 5 | 12.5 | 25 | 50 | 125 | 250 | 500 | 1250 | 2500 | 5000 |
| Pantothenic acid | 0.5 | 1 | 2 | 5 | 10 | 20 | 50 | 100 | 200 | 500 | 1000 | 2000 |
| Uridine 5'-monophosphate | 0.125 | 0.25 | 0.5 | 1.25 | 2.5 | 5 | 12.5 | 25 | 50 | 125 | 250 | 500 |
| Ribose 5-phosphate | 0.5 | 1 | 2 | 5 | 10 | 20 | 50 | 100 | 200 | 500 | 1000 | 2000 |
| Glucaric acid | 0.5 | 1 | 2 | 5 | 10 | 20 | 50 | 100 | 200 | 500 | 1000 | 2000 |
| Mevalonic acid | 0.5 | 1 | 2 | 5 | 10 | 20 | 50 | 100 | 200 | 500 | 1000 | 2000 |
| Trehalose 6-phosphate | 0.125 | 0.25 | 0.5 | 1.25 | 2.5 | 5 | 12.5 | 25 | 50 | 125 | 250 | 500 |
| Glycolic acid | 0.5 | 1 | 2 | 5 | 10 | 20 | 50 | 100 | 200 | 500 | 1000 | 2000 |
| Isonicotinic acid | 0.125 | 0.25 | 0.5 | 1.25 | 2.5 | 5 | 12.5 | 25 | 50 | 125 | 250 | 500 |
| Ethylmalonic acid | 0.25 | 0.5 | 1 | 2.5 | 5 | 10 | 25 | 50 | 100 | 250 | 500 | 1000 |
| Oxalic acid | 1.25 | 2.5 | 5 | 12.5 | 25 | 50 | 125 | 250 | 500 | 1250 | 2500 | 5000 |
| Lipoic acid | 0.125 | 0.25 | 0.5 | 1.25 | 2.5 | 5 | 12.5 | 25 | 50 | 125 | 250 | 500 |
| 2-Isopropylmalic acid | 0.125 | 0.25 | 0.5 | 1.25 | 2.5 | 5 | 12.5 | 25 | 50 | 125 | 250 | 500 |
| 3-Aminoisobutanoic acid | 0.25 | 0.5 | 1 | 2.5 | 5 | 10 | 25 | 50 | 100 | 250 | 500 | 1000 |
| Malonic acid | 0.25 | 0.5 | 1 | 2.5 | 5 | 10 | 25 | 50 | 100 | 250 | 500 | 1000 |
| Methylmalonic acid | 0.125 | 0.25 | 0.5 | 1.25 | 2.5 | 5 | 12.5 | 25 | 50 | 125 | 250 | 500 |

# Table 5.2 Regression Equations and R² Values

| Analyte | Linear Range (ng/mL) | Regression Equation | R² |
| --- | --- | --- | --- |
| L-Carnitine | 0.50000-1000.0000 | y = 0.00826 x + 4.94006e-4 | 0.997 |
| Phosphoryl choline | 0.50000-2000.0000 | y = 0.00513 x + 6.98954e-4 | 0.9988 |
| 5'-Guanylic acid | 0.25000-500.0000 | y = 0.00542 x - 5.24192e-4 | 0.9933 |
| Glucose | 25.00000-25000.0000 | y = 6.20978e-4 x - 0.00484 | 0.9917 |
| Glyceric acid | 0.50000-2000.0000 | y = 0.00141 x + 6.17212e-4 | 0.9953 |
| Galactose 1-phosphate | 0.50000-2000.0000 | y = 9.45645e-4 x + 3.36520e-4 | 0.9952 |
| Lactose | 2.50000-500.0000 | y = 3.36863e-4 x - 2.65498e-5 | 0.9969 |
| Xylose | 12.50000-50000.0000 | y = 1.40392e-4 x - 7.75957e-6 | 0.9935 |
| Galactose | 12.50000-50000.0000 | y = 3.26432e-5 x - 9.89747e-6 | 0.9923 |
| L-Rhamnose | 1.00000-2000.0000 | y = 0.00121 x + 8.56900e-6 | 0.9929 |
| Nicotinic acid | 0.50000-1000.0000 | y = 0.01402 x + 2.53028e-5 | 0.9967 |
| Glyceraldehyde | 0.50000-500.0000 | y = 0.02653 x + 0.02045 | 0.9959 |
| Gluconic acid | 5.00000-5000.0000 | y = 0.00360 x + 0.00580 | 0.9938 |
| Itaconic acid | 0.12500-50.0000 | y = 0.04775 x + 0.00113 | 0.9951 |
| cis-Aconitic acid | 0.50000-2000.0000 | y = 0.00243 x - 1.19284e-4 | 0.9926 |
| Isocitric acid | 0.50000-1000.0000 | y = 0.00107 x - 1.42315e-4 | 0.9921 |
| Succinic acid | 0.50000-2000.0000 | y = 0.01244 x + 0.00602 | 0.9947 |
| Malic acid | 0.50000-500.00000 | y = 0.00309 x + 8.48170e-4 | 0.996 |
| Adenosine 5'-monophosphate | 0.25000-1000.0000 | y = 0.00437 x + 9.62062e-4 | 0.9936 |
| 3-Phosphoglyceric acid | 5.00000-2000.0000 | y = 4.82056e-4 x - 0.00176 | 0.9914 |
| 2-Ketoglutaric acid | 1.00000-2000.0000 | y = 0.00227 x + 6.87620e-4 | 0.9979 |
| Glucose 1-phosphate | 2.00000-2000.0000 | y = 2.04121e-4 x + 2.17780e-4 | 0.9933 |
| 2-Deoxy-D-glucose | 1.00000-1000.0000 | y = 0.00234 x - 0.00108 | 0.9932 |
| Fructose | 2.00000-2000.0000 | y = 0.00279 x + 0.00294 | 0.9918 |
| Melibiose | 1.00000-2000.0000 | y = 0.00148 x + 5.85975e-5 | 0.9929 |
| L-Fucose | 1.00000-1000.0000 | y = 7.16479e-4 x + 5.72639e-6 | 0.9922 |
| Glucosamine 6-phosphate | 1.00000-2000.0000 | y = 0.00575 x - 0.00370 | 0.9936 |
| Fumaric acid | 0.50000-1000.0000 | y = 0.00323 x + 3.32397e-4 | 0.9935 |
| Fructose 6-phosphate | 1.00000-2000.0000 | y = 9.42171e-4 x - 2.69820e-4 | 0.9915 |
| Glucose 6-phosphate | 0.50000-1000.0000 | y = 0.00187 x - 1.09481e-4 | 0.9949 |
| Pyruvic acid | 1.00000-2000.0000 | y = 1.13364e-4 x + 0.00162 | 0.9977 |
| Citric acid | 25.00000-50000.0000 | y = 1.41781e-4 x - 0.00214 | 0.9942 |
| Glyoxylic acid | 1.00000-2000.00000 | y = 0.01278 x + 0.04062 | 0.9934 |
| N-acetyl-D-glucosamine | 1.25000-5000.00000 | y = 3.23087e-4 x + 7.40746e-4 | 0.9923 |
| Pantothenic acid | 0.50000-2000.00000 | y = 0.00176 x + 2.00835e-5 | 0.9992 |
| Uridine 5'-monophosphate | 0.12500-500.00000 | y = 0.00635 x + 3.08153e-4 | 0.9966 |
| Ribose 5-phosphate | 0.50000-2000.00000 | y = 1.52214e-4 x + 3.81816e-4 | 0.9942 |
| Glucaric acid | 0.50000-2000.00000 | y = 7.23487e-4 x + 9.70419e-5 | 0.9942 |
| Mevalonic acid | 1.00000-2000.00000 | y = 8.76639e-4 x + 4.45409e-4 | 0.9953 |
| Trehalose 6-phosphate | 0.12500-500.00000 | y = 0.00225 x + 2.90240e-4 | 0.9917 |
| Glycolic acid | 1.00000-2000.00000 | y = 0.00349 x + 0.00343 | 0.9923 |
| Isonicotinic acid | 0.12500-500.00000 | y = 0.01495 x + 2.08271e-4 | 0.9932 |
| Ethylmalonic acid | 0.50000-500.00000 | y = 0.00101 x - 7.84008e-5 | 0.9954 |
| Oxalic acid | 2.50000-5000.00000 | y = 1.33278e-4 x + 0.00115 | 0.995 |
| Lipoic acid | 0.25000-250.00000 | y = 0.01812 x - 6.43034e-5 | 0.9958 |
| 2-Isopropylmalic acid | 0.12500-500.00000 | y = 0.01049 x - 2.57473e-5 | 0.9956 |
| 3-Aminoisobutanoic acid | 0.50000-1000.00000 | y = 1.09892e-4 x - 3.27192e-5 | 0.9942 |
| Malonic acid | 0.25000-1000.00000 | y = 0.00703 x + 0.00160 | 0.9957 |
| Methylmalonic acid | 0.12500-500.00000 | y = 0.00231 x + 3.16794e-6 | 0.9949 |
